# Exercise Systolic Reserve and Exercise Pulmonary Hypertension Improve Diagnosis of Heart Failure With Preserved Ejection Fraction

**DOI:** 10.3389/fcvm.2022.814601

**Published:** 2022-02-09

**Authors:** Jan Verwerft, Frederik H. Verbrugge, Guido Claessen, Lieven Herbots, Paul Dendale, Andreas B. Gevaert

**Affiliations:** ^1^Heart Centre Hasselt, Jessa Hospital, Hasselt, Belgium; ^2^Faculty of Medicine and Life Sciences, Biomedical Research Institute, Hasselt University, Hasselt, Belgium; ^3^Department of Cardiovascular Diseases, Mayo Clinic, Rochester, MN, United States; ^4^Centre for Cardiovascular Diseases, University Hospital Brussels, Brussels, Belgium; ^5^Department of Cardiovascular Sciences, Katholieke Universiteit Leuven, Leuven, Belgium; ^6^Division of Cardiology, University Hospitals Leuven, Leuven, Belgium; ^7^Research Group Cardiovascular Diseases, Department GENCOR (Genetics, Pharmacology and Physiopathology of Heart, Blood Vessels and Skeleton), University of Antwerp, Antwerp, Belgium; ^8^Department of Cardiology, Antwerp University Hospital, Edegem, Belgium

**Keywords:** exercise echocardiography, diastolic stress test, HFpEF, echocardiography, cardiac imaging, cardiopulmonary exercise testing

## Abstract

**Aims:**

Diastolic stress testing (DST) is recommended to confirm heart failure with preserved ejection fraction (HFpEF) in patients with exertional dyspnea, but current algorithms do not detect all patients. We aimed to identify additional echocardiographic markers of elevated pulmonary arterial wedge pressure during exercise (exPAWP) in patients referred for DST.

**Methods and Results:**

We identified candidate parameters in 22 patients referred for exercise right heart catheterization with simultaneous echocardiography. Elevated exPAWP (≥25 mmHg) was present in 14 patients, and was best identified by peak septal systolic annular velocity <9.5 cm/s [*exS'*, area under the receiver operating characteristic curve (AUC) 0.97, 95% confidence interval 0.92–1.0] and mean pulmonary artery pressure/cardiac output slope ≥3.2 mmHg/L [mPAP/CO, AUC 0.88 (0.72–1.0)]. We propose a decision tree to identify patients with elevated exPAWP. Applying this decision tree to 326 patients in an independent non-invasive DST cohort showed that patients labeled as “high probability of HFpEF” (*n* = 85) had reduced peak oxygen uptake [13.0 (10.7–15.1) mL/kg/min, *p* < 0.001 vs. intermediate/low probability], high H2FPEF score [53 (40–72) %, *p* < 0.001 vs. intermediate/low probability], and typical clinical characteristics. The diagnostic yield of DST increased from 11% using exercise E/e', to 62% using the decision tree.

**Conclusion:**

In DST for suspected HFpEF, *exS'* was the most accurate echocardiographic parameter to identify elevated PAWP. We propose a decision tree including *exS'* and mPAP/CO for interpretation of DST. Application of this decision tree revealed typical HFpEF characteristics in patients labeled as high probability of HFpEF, and substantially reduced the number of inconclusive results.

## Introduction

Half of heart failure (HF) patients have a preserved ejection fraction (HFpEF) (1). Compared to HF with reduced ejection fraction, the diagnosis of HFpEF is often more challenging, especially when patients are not decompensated (2). Guidelines recommend using the combination of patient characteristics, natriuretic peptide levels, and echocardiography at rest to make a diagnosis of HFpEF (3, 4). However, in patients without gross volume overload who complain from chronic dyspnea, a diagnosis of HFpEF can be easily missed at rest, as many patients only develop symptoms and disproportionate elevation of cardiac filling pressures during exercise (5, 6).

Invasive hemodynamic exercise testing is considered the gold standard to rule in or rule out HFpEF based on a pulmonary arterial wedge pressure (PAWP) ≥25 mmHg or <25 mmHg during symptom-limited supine exercise (exPAWP) (7). Yet, this strategy is not broadly applied due to the invasive nature of the technique and limited expertise. A positive diastolic stress test (DST) in patients with an intermediate to high pretest probability may offer a valuable alternative to confirm the diagnosis of HFpEF, with this approach supported by a recent consensus statement of the Heart Failure Association of the European Society of Cardiology (8). DST refers to the use of echocardiography to detect impaired left ventricular (LV) diastolic functional reserve and disproportionally increased filling pressures during exercise that can result in pulmonary hypertension in many patients (9). Accordingly, elevated early mitral inflow velocity over early diastolic annular velocity (*E/e'*) and tricuspid regurgitation (TR) velocity during exercise (*exE/e'*, exTR) are used to support a diagnosis of HFpEF (10–12). Different algorithms have been proposed incorporating *exE/e'*, exTR and/or resting echo variables (8, 10, 12). Invasive validation has only been performed for *exE/e'*, of which the positive predictive value is good at 85–93%, but the low negative predictive value (55–77%) results in a substantial amount of false negative tests (12).

The aim of this study was to identify additional echocardiographic markers of elevated PAWP ≥25 mmHg, assessed by gold-standard invasive haemodynamic exercise testing with simultaneous echocardiography and cardiopulmonary exercise test (CPET), performed because of unexplained exertional dyspnea. Subsequently, we aimed to apply these echocardiographic parameters in patients referred for non-invasive DST with simultaneous CPET.

## Methods

### Study Population

**Figure 1:**
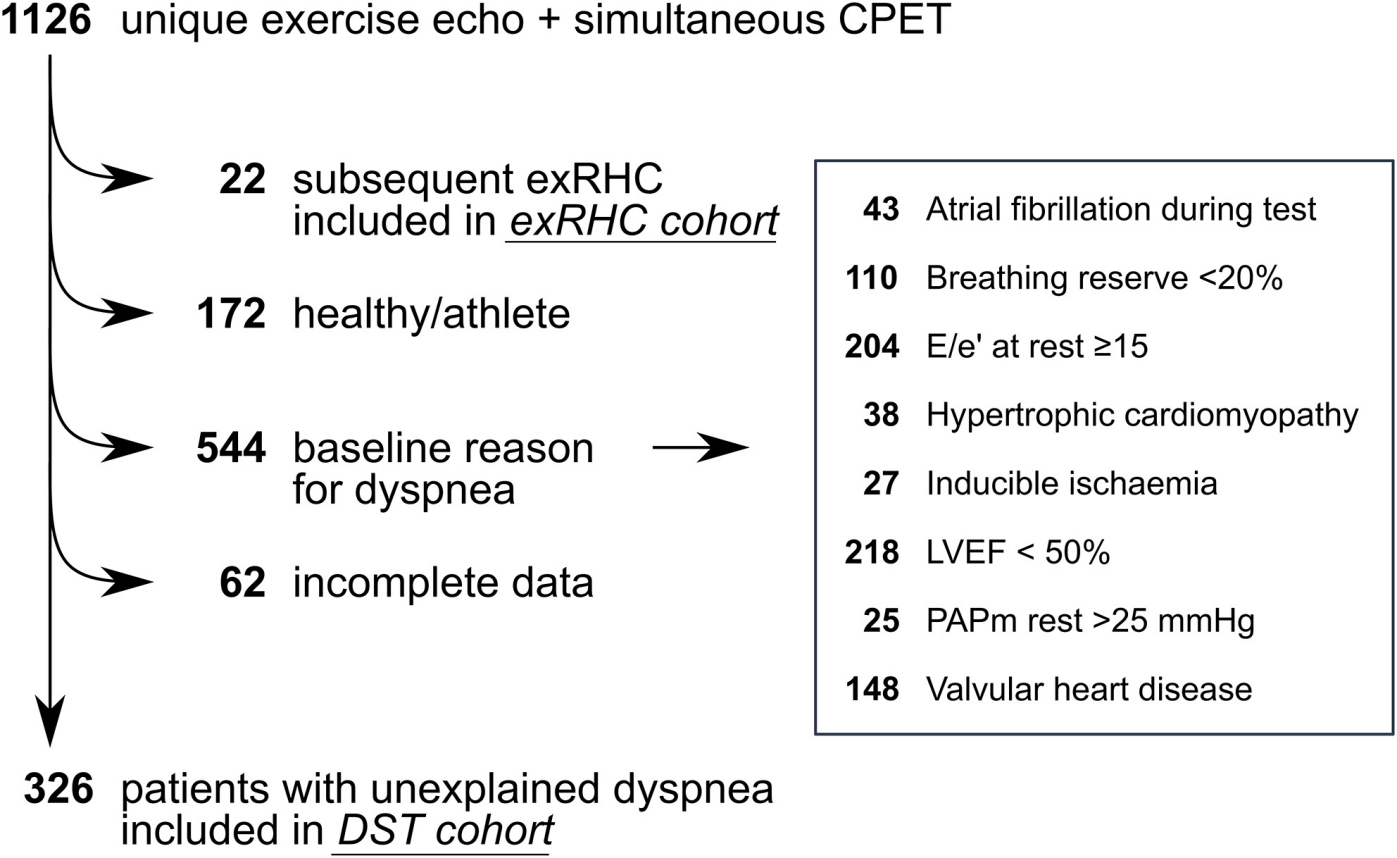
Study flow. We screened 1,126 unique patients who underwent simultaneous exercise echocardiography and cardiopulmonary exercise test. Patients who underwent subsequent exRHC (*n* = 22) were included in the exRHC cohort. Patients with unexplained dyspnea were included in the DST cohort (*n* = 326). We excluded healthy subjects (*n* = 172), patients with incomplete data (*n* = 62) and patients with a baseline reason for dyspnea (*n* = 544). Note that some patients had more than 1 reason for dyspnea. Valvular heart disease was defined as more than mild valvular stenosis, more than moderate left-sided valvular insufficiency, or previous valvular surgery. DST, diastolic stress test; exRHC, exercise right heart catheterization; LVEF, left ventricular ejection fraction; PAPm, mean pulmonary artery pressure.

We performed a retrospective analysis of patients referred to Jessa Hospital (Hasselt, Belgium) because of exertional dyspnea not sufficiently explained by resting examinations. We screened patients referred from April 2017 to May 2020 (Figure 1). We excluded healthy subjects including athletes, patients with incomplete data, and patients with another explanation for dyspnea: left ventricular ejection fraction (LVEF) <50%, current atrial fibrillation, pulmonary limitation to exercise (defined as peak ventilation >80% of maximal voluntary ventilation), E/e' >15 at rest, hypertrophic cardiomyopathy, inducible myocardial ischemia, pulmonary hypertension at rest, or valvular heart disease (defined as more than mild valvular stenosis, more than moderate left-sided valvular insufficiency, or previous valvular surgery). Non-invasive DST was performed in all consecutive patients (DST cohort). If non-invasive DST was inconclusive, patients were offered invasive hemodynamic exercise testing with simultaneous echocardiography and gas exchange measurement (exRHC cohort). We used the exRHC cohort for derivation of the echocardiographic variables associated with elevated PAWP. We applied these novel variables to the DST cohort. Patients included in the exRHC cohort were excluded from validation analyses in the DST cohort. This study complies with the Declaration of Helsinki and was approved by the Ethical Committee of the Jessa Hospital. All patients provided informed consent.

### Study Protocol

All patients underwent CPET with respiratory gas analysis (CS-200, Schiller). Exercise was performed on a semi-supine bicycle ergometer (ErgoLine) with a continuous ramp protocol aimed for a total exercise duration of 10–12 min. In all patients, echocardiography data was simultaneously collected during 2 stage holds, at submaximal level (aerobic threshold) and at peak exercise, as described previously (13, 14). In the exRHC cohort, additionally a pulmonary artery catheter (Edwards Lifesciences) was placed under fluoroscopic guidance at the catheterization lab before start of the CPET and the right radial artery was cannulated with a 5F arterial catheter, to obtain arterial and mixed venous blood gas samples and measure PAWP.

Invasive, CPET, and echocardiographic measurements are described in detail in the Supplementary Methods. For echocardiography, peak mitral systolic annular velocity (*S'*) was measured using color tissue Doppler imaging (TDI) at the septal mitral annulus (Supplementary Figure 1). E/e' was also measured at the septal mitral annulus. Colloid enhancement of the tricuspid insufficiency signal was systematically employed to measure systolic pulmonary artery pressure (sPAP), as previously described (15). Cardiac output (CO) was measured using the left ventricular outflow tract method.

### Definitions and Thresholds

Elevated cardiac filling pressures were primarily defined as a peak exercise PAWP ≥25 mmHg on invasive hemodynamic assessment, and alternatively as PAWP/CO slope ≥2.0 mmHg/L (5, 16). Exercise pulmonary hypertension was defined as mean pulmonary artery pressure over CO (mPAP/CO) slope ≥3.0 mmHg/L by invasive hemodynamic assessment, and ≥3.2 mmHg/L by echocardiography, as previous studies reported higher values on echocardiography (15).

Diagnosis of HFpEF on non-invasive DST was considered highly probable when septal *exE/e'* was ≥15 (12). As a sensitivity analysis, we also applied the most recent American Society of Echocardiography (ASE) and European Association of Cardiovascular Imaging (EACVI) recommendations (high probability when septal *exE/e'* ≥15, exTR >2.8 m/s and resting *e'* <7 cm/s, low probability when septal *exE/e'* <10 and exTR <2.8 m/s, inconclusive when not meeting either criteria) (10, 11).

To evaluate the performance of the novel echocardiographic markers of elevated exPAWP, the probability of HFpEF according to the novel marker was compared to surrogate HFpEF indicators: peak oxygen uptake (VO_2_) and logistic H2FPEF score. The latter calculates the probability of HFpEF through clinical and echocardiographic parameters, and has been developed using invasive exRHC measurements (17).

### Statistical Analysis and Sample Size Calculation

Detailed methods for statistical analysis and sample size calculations are described in the Supplementary Methods. In summary, DST parameters were compared between patients with elevated vs. normal exPAWP using Mann-Whitney *U*-test (single measurement during DST, for example mPAP/CO slope) or linear mixed models (repeated measurement during DST, for example *E/e'*). Linear mixed models were constructed using patient number as random factor, and exercise, elevated exPAWP, and their interaction as fixed factors. For each DST parameter with potential to identify elevated exPAWP, a receiver operating characteristic curve was determined, and area under the curve (AUC) was calculated with the trapezoidal rule. Ninety-five percentage confidence intervals (CI) were calculated using stratified bootstrap replicates. AUC were compared using Delong's test.

## Results

### Population

We screened 1,126 patients, of whom 326 patients had unexplained dyspnea and were included in the DST cohort, and 22 patients were subsequently referred for exRHC (Figure 1). In the exRHC cohort, 16 patients were referred because of an inconclusive DST (not meeting inclusion nor exclusion criteria for HFpEF), and 6 were referred because of discrepancy between *exE/e'* and exTR on DST. Compared to the exRHC cohort, patients in the DST cohort had a lower prevalence of coronary artery disease, but otherwise similar baseline characteristics (Table 1).

**Table 1:** Baseline characteristics of the study populations.

**Characteristic**	**ExRHC cohort** **(*n* = 22)**	**DST cohort** **(*n* = 326)**	***P*-value**
Age, years	65 (57–71)	66 (56–72)	0.601
Women	10 (45)	174 (53)	0.617
Heart rate, bpm	68 (62–72)	69 (63–80)	0.577
Systolic blood pressure, mmHg	150 (127–155)	139 (124–150)	0.256
BMI, kg/m^2^	27.5 (26.1–30.9)	26.9 (24.0–30.0)	0.282
**Past medical history**			
Atrial fibrillation	5 (23)	57 (17)	0.738
Coronary heart disease	8 (36)	54 (17)	**0.039**
Diabetes	3 (14)	40 (12)	0.999
Hypertension	11 (50)	160 (49)	0.999
**Medication use**			
ACE inhibitor or ARB	6 (27)	89 (37)	0.520
Aldosterone antagonist	4 (18)	27 (12)	0.613
Beta blocker	11 (50)	102 (42)	0.592
Calcium antagonist	4 (18)	46 (20)	0.999
Diuretic	5 (23)	38 (17)	0.708
Nitrate	2 (9)	13 (6)	0.862
**Laboratory analysis**			
Hemoglobin, g/dL	13.8 (12.7–15.0)	13.9 (12.9–14.8) *(n = 224)*	0.181
EGFR, mg/dL	72 (67–80) *(n = 16)*	76 (61–91) *(n = 198)*	0.802

*Continuous variables: median (IQR), P-value from Mann-Whitney U-test. Categorical variables: no. (%), P-value from Chi-square test. ACE, angiotensin conversion enzyme; ARB, angiotensin receptor blocker; BMI, Body mass index; EGFR, Estimated glomerular filtration rate using CKD-EPI formula. Bold type: p < 0.05, Italic type: number of available measurements when smaller than group size*.

### Derivation of Peak Exercise S' as Surrogate for Elevated Cardiac Filling Pressures

In the exRHC cohort, PAWP ≥25 mmHg during exercise was recorded in 14 patients, while 8 patients had normal exPAWP. Comparison of baseline characteristics revealed older age, lower heart rate, more beta blocker use, and worse renal function in patients with elevated exPAWP (all *p* < 0.05, Supplementary Table 1).

**Figure 2:**
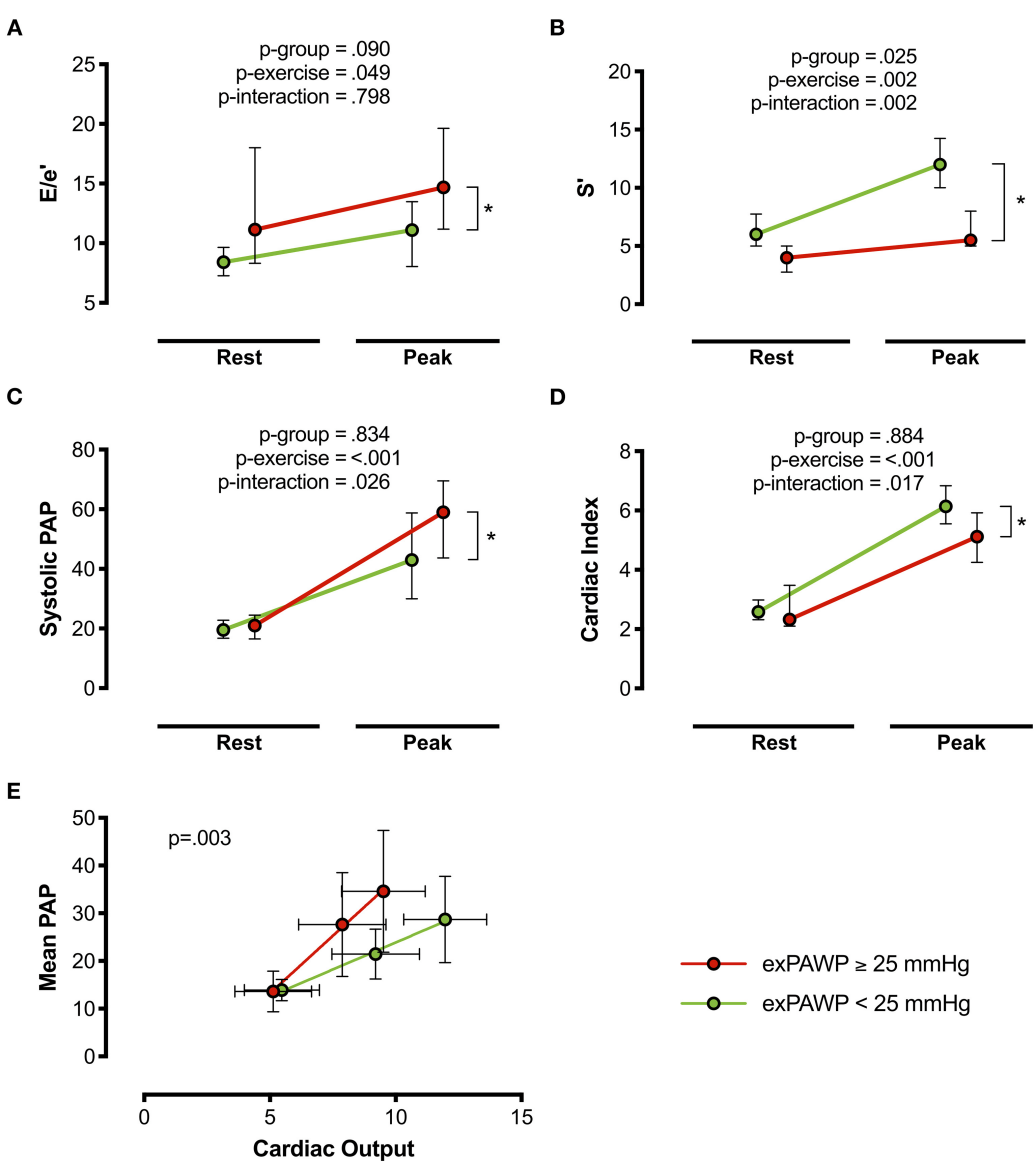
DST parameters associated with elevated exPAWP. Results of non-invasive septal E/e' **(A)**, S' **(B)**, systolic PAP **(C)**, cardiac index **(D)**, and mean PAP/CO slope **(E)** in the exRHC cohort at rest and peak exercise. Red: patients with elevated exPAWP (*n* = 14), green: patients with normal exPAWP (*n* = 8). *P*-values from linear mixed models. *p < 0.05 in multiplicity-adjusted comparison of peak values. exPAWP, pulmonary artery wedge pressure during peak exercise; PAP; pulmonary artery pressure.

Among echocardiographic parameters, peak exercise septal systolic velocity (*exS'), exE/e'*, peak sPAP, mPAP/CO slope, peak cardiac index, and rest LV mass index were associated with elevated exPAWP (Figure 2; Supplementary Table 2). Among these echocardiographic parameters, no strong correlations were demonstrated (Supplementary Table 3).

*ExS'* was the best echocardiographic parameter associated with elevated exPAWP, with an AUC of 0.97 (CI 0.92–1.0), compared to 0.88 (CI 0.72–1.0) for mPAP/CO slope, 0.79 (CI 0.58–0.99) for peak cardiac index, 0.76 (CI 0.55–0.96) for *exE/e'*, and 0.76 (CI 0.54–0.97) for peak sPAP (Supplementary Figure 2). *ExS'* had a significantly higher AUC compared to *exE/e'* (*p* = 0.039) and peak sPAP (*p* = 0.035), but not to mPAP/CO slope (*p* = 0.239) or peak cardiac index (*p* = 0.099).

A threshold of *exS'* <9.5 cm/s had a specificity of 88% and sensitivity of 100% for detecting exPAWP ≥25 mmHg. *ExE/e'* ≥15 had a specificity of 100% and sensitivity of 50%; mPAP/CO slope ≥3.2 mmHg/L had a specificity of 63% and sensitivity of 85%.

As a sensitivity analysis, elevated cardiac filling pressures were alternatively defined as PAWP/CO slope >2.0 mmHg/L. AUC were comparable to the standard definition for *exS'* (0.94, CI 0.84–1.0), *exE/e'* (0.85, CI 0.68–1.0), peak sPAP (0.72, CI 0.46–0.98), and mPAP/CO slope (0.72, CI 0.46–0.97), but lower for peak cardiac index (0.44, CI 0.17–0.70).

### Decision Tree for Determining Probability of HFpEF in DST

**Figure 3:**
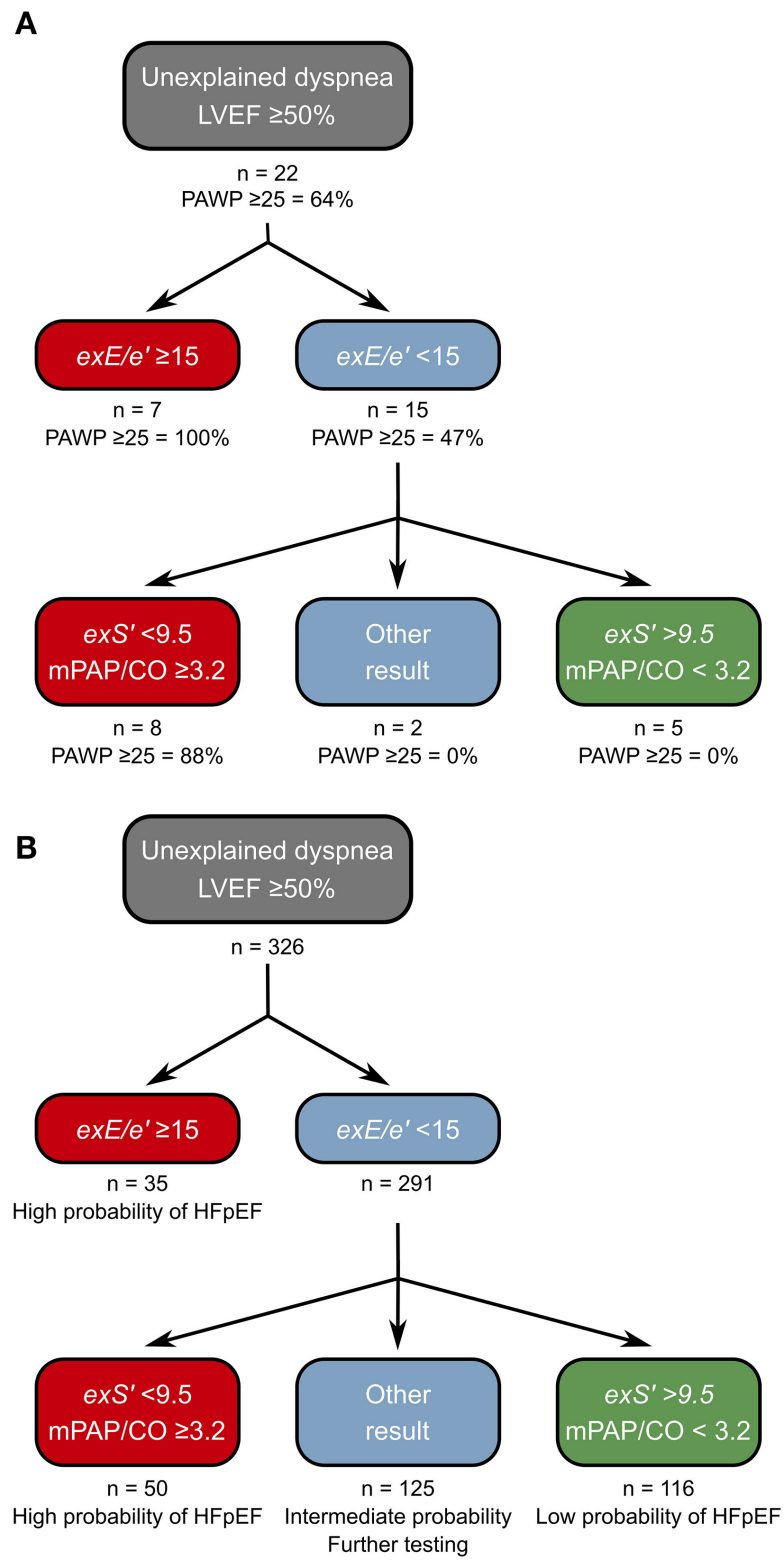
Proposed decision tree for diagnosis of HFpEF on DST. **(A)** Derivation of the decision tree in the exRHC cohort. Step 1: the existing approach using *exE/e'* is maintained. Step 2: *exS'* and mPAP/CO slope are determined, HFpEF is considered high probability if *exS'* <9.5 cm/s and mPAP/CO slope ≥3.2 mmHg/L, and low probability if both parameters below these thresholds. Thus, all patients with exPAWP ≥25 mmHg are identified. A single patient is false positive using this approach. **(B)** Application of the decision tree to the non-invasive DST cohort. Of 291 patients with normal *exE/e'*, 116 (40%) had *exS'* >9.5 cm/s and mPAP/CO slope <3.2 mmHg/L, we propose that probability of HFpEF is low in these patients. A total of 50 patients (17%) had low *exS'* and elevated mPAP/CO slope, we propose that probability of HFpEF is high in these patients. In the remaining 125 patients, we propose to perform additional investigations before establishing a diagnosis of HFpEF. CO, cardiac output; *exE/e'*, highest septal E/e' recorded during exercise; *exS'*, S' at peak exercise; mPAP, mean pulmonary artery pressure.

In the exRHC cohort, 7/22 patients had a positive DST (*exE/e'* ≥15). All these patients indeed had exPAWP ≥25 mmHg. Thus, 8 patients remained with elevated exPAWP and normal *exE/e'*. However, all 14 patients with elevated exPAWP had *exS'* <9.5 cm/s. A decision tree consisting of *exE/e'* in a first step and low *exS'* in a second step (Figure 3A), would successfully identify all patients with elevated exPAWP, at the cost of 1 false positive patient (exPAWP = 23 mmHg).

Most patients with clinically relevant HFpEF exhibit pulmonary hypertension during exercise (18). Indeed, all patients with *exS'* <9.5 and PAWP ≥25 mmHg had mPAP/CO slope ≥3.2 mmHg/L. Moreover, mPAP/CO slope was the second best parameter in the AUC analysis. Thus, we suggest an algorithm based on a first step assessing *exE/e'*, adding *exS'* and mPAP/CO slope in a second step (Figures 3A,B).

### Applying the Decision Tree in the DST Cohort

In the DST cohort, using *exE/e'* ≥15 a diagnosis of HFpEF was made in 35 out of 326 patients (11%). A total of 291 patients (89%) remained (Figure 3B). Applying the stricter ASE/EACVI recommendations, the majority of patients had inconclusive results (294 patients, 90%, Supplementary Figure 3). Among the 291 patients with normal *exE/e'*, 155 patients (53%) had *exS'* <9.5 cm/s, 64 patients (22%) had mPAP/CO slope ≥3.2 mmHg/L, and 50 patients (17%) had both. Also, 116 patients (40%) had normal values for both *exS'* and mPAP/CO. Most of the patients with elevated *exE/e'* had abnormal *exS'* (32 patients, 86%).

**Figure 4:**
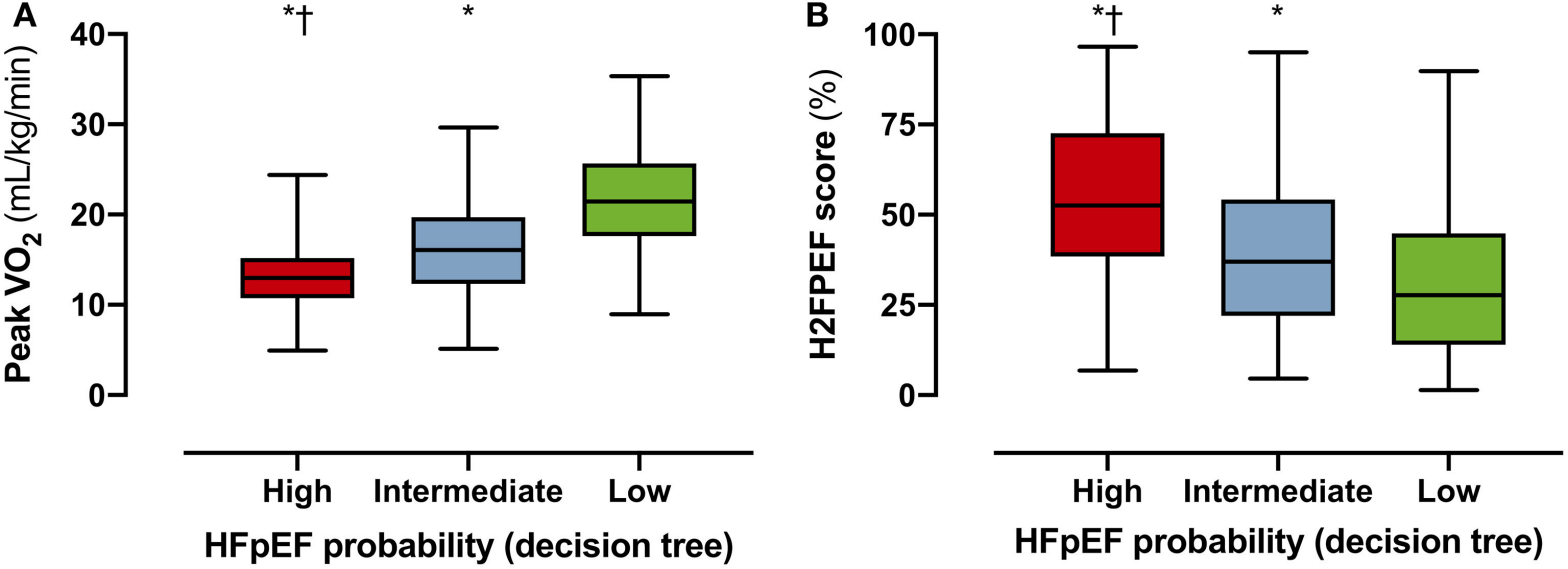
Performance of decision tree in the DST cohort. To evaluate the performance of the decision tree (Figure 3), the probability of HFpEF according to the decision tree (high/intermediate/low) was compared to surrogate HFpEF indicators peak VO_2_ **(A)** and logistic H2FPEF score **(B)**, which calculates the probability of elevated exPAWP in percentage through clinical and echocardiographic parameters (17). Multiplicity-corrected *P-*values from Kruskal-Wallis test. HFpEF, heart failure with preserved ejection fraction; Peak VO_2_, peak oxygen uptake, *p < 0.001 compared to low; *p* < 0.001 compared to intermediate.

Applying the proposed decision tree, 166 patients (57% of inconclusive tests) could be reclassified as “high probability of HFpEF” or “low probability of HFpEF,” reducing the number of inconclusive tests from 291 (89%) to 125 (43%). Patients in the “high probability” group had a worse exercise capacity compared to patients with intermediate or low probability: lower peak VO_2_ (Figure 4A), lower peak heart rate, lower workload, and steeper ventilation over carbon dioxide production slope (Table 2). Patients classified as “high probability” had a higher logistic H2FPEF score compared to patients with intermediate or low probability, indicating high likelihood of elevated exPAWP (Figure 4B; Table 2). Patients in the “high probability” group were older, more frequently had atrial fibrillation, and had worse renal function compared to patients with intermediate or low probability (Table 2). Finally, compared to the other groups, patients classified as “high probability” had higher resting *E/e'*, higher *exE/e'* and exercise sPAP, and reduced peak cardiac index (Table 2).

**Table 2:** Clinical characteristics and measurements in DST cohort, stratified according HFpEF probability (decision tree).

	**High probability (*n* = 85)**	**Intermediate probability (*n* = 125)**	**Low probability (*n* = 116)**	***P*-value**
**Surrogate HFpEF indicators**				
H2FPEF score, %	53 (40–72)^*^^†^	37 (22–54)^*^	28 (14–45)	**<0.001**
Peak VO_2_, mL/kg/min	13.0 (10.7–15.1)^*^^†^	16.0 (12.2–19.4)^*^	21.4 (17.6–25.6)	**<0.001**
**Clinical characteristic**				
Age, years	72 (67–78)^*^^†^	66 (59–71)^*^	59 (50–66)	**<0.001**
Women	59 (69)^*^	84 (67)^*^	31 (27)	**<0.001**
Heart rate, bpm	68 (61–76)^*^	68 (64–79)	71 (65–84)	**0.026**
Systolic blood pressure, mmHg	144 (128–156)	139 (123–152)	135 (123–148)	0.109
BMI, kg/m^2^	27.3 (24.1–29.9)	26.8 (23.9–30.0)	26.8 (24.2–30.0)	0.894
**Past medical history**				
Atrial fibrillation	23 (27)^*^	20 (16)	14 (12)	**0.022**
Coronary heart disease	14 (16)	17 (14)	23 (20)	0.441
Diabetes	10 (12)	17 (14)	13 (11)	0.868
Hypertension	56 (67)^*^	62 (50)	42 (36)	**<0.001**
**Laboratory analysis**				
Hemoglobin, g/dL	13.7 (12.6–14.6) *(n = 63)*	13.8 (12.9–14.6) *(n = 85)*	14.3 (13.0–15.2) *(n = 76)*	0.065
EGFR, mg/dL	65 (54–73)^*^^†^ *(n = 54)*	80 (62–91)^*^(n = 76)	86 (72–97) *(n = 68)*	**<0.001**
**Echocardiography: rest**				
*E/e'* septal, ratio	11.8 (10.0–13.3)^*^^†^	10.0 (8.2–11.5)^*^	9.0 (7.5–10.5)	**<0.001**
*S'*, cm/s	4.0 (3.8–5.0)^*^	4.5 (3.1–6.0)^*^	6.0 (5.0–8.0)	**<0.001**
Systolic PAP, mmHg	22 (20–25)^*^	22 (19–25)	20 (18–24)	**0.009**
LV ejection fraction, %	63 (57–70)	62 (56–69)	62 (57–67)	0.613
Cardiac index, L/m^2^	2.4 (1.9–2.9)	2.4 (2.1–2.8)	2.6 (2.2–3.2)	**0.048**
LV mass index, g/m^2^	89 (70–106)	80 (65–96)	85 (64–101)	0.087
Left atrial volume index, mL/m^2^	24 (19–33)^*^^†^ *(n = 61)*	20 (16–29)^*^(n = 90)	17 (12–23) *(n = 96)*	**<0.001**
RVFAC, %	47 (41–55) *(n = 74)*	50 (42–56) *(n = 101)*	50 (43–57) *(n = 101)*	0.234
**Echocardiography: peak exercise**				
*E/e'* septal, ratio^‡^	14.4 (12.2–16.7)^*^^†^	10.7 (9.2–12.6)^*^	9.5 (8.3–10.8)	**<0.001**
*S'*, cm/s	7.0 (5.0–8.0)^*^^†^	8.0 (6.6–9.0)^*^	11.0 (10.0–13.0)	**<0.001**
Systolic PAP, mmHg	50 (45–56)^*^^†^	45 (40–50)	43 (40–49)	**<0.001**
LV ejection fraction, %	68 (60–75)^*^	68 (62–74)^*^	70 (66–76)	**0.009**
Cardiac index, L/min/m^2^	4.7 (4.0–5.4)^*^^†^	5.6 (4.8–6.3)^*^	6.6 (5.5–7.2)	**<0.001**
Mean PAP/CO slope, mmHg/L/min	4.1 (3.4–5.1)^*^^†^	2.3 (1.9–2.8)^*^	2.0 (1.4–2.4)	**<0.001**
RVFAC, %	56 (48–61) *(n = 73)*	56 (50–62) *(n = 109)*	57 (50–65) *(n = 101)*	0.228
**Cardiopulmonary exercise test**				
Peak heart rate, bpm	111 (97–119)^*^^†^	121 (110–138)^*^	137 (122–153)	**<0.001**
Workload, W	70 (53–85)^*^^†^	86 (69–112)^*^	137 (100–161)	**<0.001**
VE/VCO_2_ slope, unitless	30.8 (28.2–34.4)^*^^†^	28.5 (25.6–31.7)^*^	26.6 (24.8–29.0)	**<0.001**
CO/VO_2_ slope, unitless	5.3 (4.2–6.8)^†^	6.4 (5.0–7.6)^*^	5.5 (4.5–6.7)	**<0.001**

*See Figure 3B for decision tree. Continuous variables: median (IQR), P-value from Kruskal-Wallis test. Categorical variables: no. (%), P-value from Chi-square test. BMI, Body mass index, CO, cardiac output, EGFR, Estimated glomerular filtration rate using CKD-EPI formula, H2FPEF score, score estimating likelihood of heart failure with preserved ejection fraction based on (17), LV, left ventricular, PAP, pulmonary artery pressure, RVFAC, right ventricular fractional area change, VE, ventilation, VCO_2_, carbon dioxide removal, VO_2_, oxygen uptake*,

* *p < 0.05 vs. Low probability*,

† *p < 0.05 vs. Intermediate probability*,

‡ *Highest septal E/e' value obtained during entire duration of exercise. Bold type: p < 0.05, Italic type: number of available measurements when smaller than group size*.

Supplementary Figure 4 shows the percentage of true and false positive tests using different DST criteria for diagnosis of HFpEF. All current criteria show a lack of sensitivity: of patients with invasively proven HFpEF, ASE/EACVI recommendations detected 43%, the Heart Failure Association consensus on HFpEF 21%, and *exE/*e' alone 50%. The decision tree proposed in this paper detected 100% of HFpEF patients, at the cost of 13% false positives.

### Reproducibility of DST Parameters

*ExS'* was measured successfully in all patients in the exRHC cohort, and in 315 patients (97%) in the DST cohort. *ExS'* was highly reproducible, with an interobserver agreement of 0.97 (CI 0.92–0.99). Measurement of mPAP/CO was successful in 325 patients (99%) and showed good interobserver agreement of 0.73 (CI 0.53–0.87). In comparison, *exE/e'* could be measured in all patients and had an interobserver agreement of 0.83 (CI 0.69–0.92). Bland-Altman plots are provided in Supplementary Figure 5.

## Discussion

In this study, we established septal *exS'* and mPAP/CO slope as compelling parameters to improve identification of elevated cardiac filling pressures in a small cohort of patients referred for simultaneous exRHC and DST. A threshold of *exS'* <9.5 cm/s had a high sensitivity and specificity to identify exPAPW ≥25 mmHg. We propose a decision tree to diagnose HFpEF on DST, incorporating *exS'* and mPAP/CO slope. Applying this decision tree to 326 patients with unexplained dyspnea substantially improved the diagnostic yield of DST from 11% (using guideline recommendations) to 57% (using the decision tree).

Current ASE/EACVI recommendations recommend the use of *exE/e'* and sPAP to diagnose HFpEF on DST (10, 11). These recommendations are based on early studies focusing solely on *exE/e'*, disregarding other possible correlates of elevated exPAWP (19). Most of these were performed without concurrent exPAWP measurement, and subsequent invasive validation studies showed at most a moderate correlation between *exE/e'* and PAPW (12, 20). Another limitation of the evaluation of *exE/e'* relates to the influences of increased respiratory rate and tachycardia that occur during exercise. Hence, fusion of E/A waves and e'/a' waves often occurs beyond heart rates of 100 bpm, thereby compromising the accuracy of this assessment. E and e' are also highly load dependent, which results in a large variability at peak exercise, when the increased respiratory rate induces shifts in preload and afterload (21, 22). *ExE/e'* has a good positive predictive value for diagnosis of elevated exPAWP, but its negative predictive value (55–77%) allows a substantial amount of false negative results (12, 19).

A recent Heart Failure Association expert consensus paper proposed DST in patients with an intermediate to high pre-test probability of HFpEF (8). Compared to the ASE/EACVI recommendations, the authors removed the resting echo criteria but included a stricter cutoff of >3.4 m/s for exercise TR. In our exRHC cohort, this approach showed reduced sensitivity for the diagnosis of HFpEF compared to ASE/EAVCI recommendations (Supplementary Figure 4).

It is well-accepted that patients with HFpEF not only have impaired diastolic cardiac function, but also suffer from subtle reductions in systolic function despite a normal LVEF (23, 24). Measurements of longitudinal function, such as strain and strain rate, have emerged as less afterload dependent surrogates of systolic function, but are affected by respiratory variation in image quality at peak exercise. In contrast, systolic velocity of the mitral annulus (*S'*) can be easily obtained at peak exercise regardless of heart rate and image quality (in 96% of patients in our study), while showing high reproducibility. From a mechanistic point of view, the reduction of *exS'* in patients with increased exPAWP during exercise may be explained by decreased diastolic suction and elastic recoil resulting from a lack of systolic functional reserve. Hence, as the capacity of the LV to decrease its end-systolic volume during exercise is reduced, the driving force for early diastolic suction to enable is impaired and rapid LV filling becomes exquisitely dependent on increased filling pressures across the mitral valve.

Other studies have previously evaluated longitudinal LV function during exercise in HFpEF patients. Wang et al. found reduced values of resting *S'* and *exS'* in HFpEF patients compared to controls (25). *ExS'* correlated well-with peak VO_2_ (26), and was a significant predictor of all-cause mortality and HF hospitalization (27).

Because each individual echo parameter has its limitations, including *exS'*, we firmly believe the diagnosis of HFpEF should not be based on a single parameter. Thus, we proposed a decision tree incorporating several parameters. Using our proposed decision tree (Figure 3B), 57% of patients with normal *exE/e'* could be reclassified as high or low probability of HFpEF, substantially improving the diagnostic yield of DST. In the decision tree, we maintain *exE/e'* in the first step because of its extensive validation in multiple populations, and its high specificity (12, 19, 28). In a next step, *exS'* and mPAP/CO slope are evaluated. HFpEF is considered high probability when *exS'* <9.5 cm/s and mPAP/CO slope ≥3.2 mmHg/L, and low probability when both are below these thresholds, based on our current findings. In our opinion, an indication of exercise pulmonary hypertension must be present for the diagnosis of HFpEF using DST alone, because of the close pathophysiological relation between left atrial pressure, PAWP and mPAP. We chose mPAP/CO slope rather than sPAP, because (1) mPAP/CO slope is more accurate in situations where peak exercise CO is abnormal, such as in HFpEF (15), (2) mPAP/CO was the next-to-best parameter in the AUC analysis, (3) in the exRHC cohort all patients with *exS'* <9.5 cm/s and elevated exPAWP had a mPAP/CO slope above threshold, and (4) pulmonary vascular dysfunction is a known predictor of adverse outcomes in HFpEF (29).

Importantly, the proposed decision tree incorporates several aspects of HFpEF pathophysiology, including elevated filling pressures during exercise (*exE/e', exS'*), longitudinal LV function (*exS'*), LV relaxation (*e'*) and exercise pulmonary hypertension (mPAP/CO).

In a number of patients, exercise pulmonary hypertension was not present, but *exS'* <9.5 cm/s indicated elevated exPAWP. This may reflect early HFpEF in patients with relatively compliant left atrium and pulmonary vasculature, underestimation of mPAP/CO slope on DST, or lower specificity of *exS'* in an unselected population. In these cases, other methods can aid to establish a final diagnosis of HFpEF. The gold standard investigation for these patients remains an exRHC, as sPAP and mPAP/CO slope are generally underestimated on echocardiography when compared to invasive measurement (15).

Besides the obvious clinical impact on the HFpEF diagnostic process, our results also have implications for HFpEF clinical trials. Inclusion criteria of HFpEF clinical trials thus far included only echocardiography measurements at rest. An improved diagnostic yield of DST as suggested in our results, reducing the need for backup invasive haemodynamic exercise testing, could pave the way for DST as inclusion criterion for HFpEF clinical trials.

Our study results should be interpreted in the context of some limitations. Color TDI is angle-dependent, however the use of offline repositioning and the use of septal rather than lateral *S'* mitigated the impact of this limitation. Conventional pulse wave TDI was used for *e'* measurement. Whether using a pulse wave TDI signal optimized for assessing *S'* has equal diagnostic capabilities, remains to be studied.

A “gold standard” to diagnose HFpEF non-invasively is currently still lacking. As such, we used several surrogate measures (peak VO_2_, logistic H2FPEF score) and supporting features (diastolic function, typical clinical characteristics) in the DST cohort to demonstrate differences between patients classified as high, intermediate or low probability of HFpEF.

The absence of systematic natriuretic peptide measurement precludes a full comparison of the findings in the DST cohort with the HFA consensus criteria.

Due to the relatively high prevalence of coronary artery disease in the exRHC cohort, our findings should be interpreted with caution in other populations. None of the exRHC cohort patients had evidence of inducible myocardial ischemia or wall motion abnormalities in the basal inferoseptum.

Furthermore, the small sample size of the exRHC cohort compared to the DST cohort suggests a highly selected population. Our results should be validated in a larger patient cohort.

We conclude that *exS'* was the most accurate parameter to identify patients with elevated cardiac filling pressures in a cohort of patients referred for exRHC because of exertional dyspnea. We propose a decision tree to diagnose elevated exPAWP on DST in patients with unexplained dyspnea and LVEF ≥50%. Applying this decision tree for the diagnosis of HFpEF on DST substantially improved the diagnostic yield from 11% (using *exE/e'* alone) to 62% (using the decision tree). Validation in a separate exRHC cohort is desirable before application of our findings in clinical practice.

## Data Availability Statement

The raw data supporting the conclusions of this article will be made available by the authors, without undue reservation.

## Ethics Statement

The studies involving human participants were reviewed and approved by Ethisch Comité Jessa Ziekenhuis Hasselt. Written informed consent for participation was not required for this study in accordance with the national legislation and the institutional requirements.

## Author Contributions

JV and AG contributed to the conception or design of the work. AG drafted the manuscript. All authors contributed to the acquisition, analysis, interpretation of data for the work, critically revised the manuscript, gave final approval, agreed to be accountable for all aspects of work ensuring integrity, and accuracy.

## Funding

FV is supported by the Special Research Fund (BOF) of Hasselt University (BOF19PD04). GC is supported by the Frans Van De Werf Fund for Clinical Cardiovascular Research and the Mathilde Horlait-Dapsens Scholarship.

## Conflict of Interest

The authors declare that the research was conducted in the absence of any commercial or financial relationships that could be construed as a potential conflict of interest.

## Publisher's Note

All claims expressed in this article are solely those of the authors and do not necessarily represent those of their affiliated organizations, or those of the publisher, the editors and the reviewers. Any product that may be evaluated in this article, or claim that may be made by its manufacturer, is not guaranteed or endorsed by the publisher.

## References

[1] Dunlay SM Roger VL Redfield MM. Epidemiology of heart failure with preserved ejection fraction. Nat Rev Cardiol. (2017) 14:591–602. 10.1038/nrcardio.2017.65 28492288

[2] Van Riet EES Hoes AW Limburg A Landman MAJ Van Der Hoeven H Rutten FH. Prevalence of unrecognized heart failure in older persons with shortness of breath on exertion. Eur J Heart Fail. (2014) 16:772–7. 10.1002/ejhf.110 24863953

[3] Ponikowski P Voors AA Anker SD Bueno H Cleland JGF Coats AJS. 2016 ESC Guidelines for the diagnosis and treatment of acute and chronic heart failure. Eur Heart J. (2016) 37:2129–200. 10.1093/eurheartj/ehw128 27206819

[4] Yancy CW Jessup M Bozkurt B Butler J Casey DE Colvin MM. 2017 ACC/AHA/HFSA focused update of the 2013 ACCF/AHA guideline for the management of heart failure. J Am Coll Cardiol. (2017) 70:776–803. 10.1016/j.jacc.2017.04.025 28461007

[5] Borlaug BA Nishimura RA Sorajja P Lam CSP Redfield MM. Exercise hemodynamics enhance diagnosis of early heart failure with preserved ejection fraction. Circ Heart Fail. (2010) 3:588–95. 10.1161/CIRCHEARTFAILURE.109.930701 20543134 PMC3048586

[6] Xu B Klein AL. Utility of echocardiography in heart failure with preserved ejection fraction. J Card Fail. (2018) 24:397–403. 10.1016/j.cardfail.2018.05.005 29802895

[7] Esfandiari S Wolsk E Granton D Azevedo L Valle FH Gustafsson F. Pulmonary arterial wedge pressure at rest and during exercise in healthy adults: a systematic review and meta-analysis. J Card Fail. (2019) 25:114–22. 10.1016/j.cardfail.2018.10.009 30366054

[8] Pieske B Tschöpe C de Boer RA Fraser AG Anker SD Donal E. How to diagnose heart failure with preserved ejection fraction: the HFA–PEFF diagnostic algorithm: a consensus recommendation from the heart failure association (HFA) of the European society of cardiology (ESC). Eur Heart J. (2019) 40:3297–317. 10.1093/eurheartj/ehz641 31504452

[9] Borlaug BA Kane GC Melenovsky V Olson TP. Abnormal right ventricular-pulmonary artery coupling with exercise in heart failure with preserved ejection fraction. Eur Heart J. (2016) 37:3293. 10.1093/eurheartj/ehw241 27354047 PMC8483148

[10] Lancellotti P Pellikka PA Budts W Chaudhry FA Donal E Dulgheru R. The clinical use of stress echocardiography in non-ischaemic heart disease: recommendations from the European association of cardiovascular imaging and the American society of echocardiography. Eur Heart J Cardiovasc Img. (2016) 17:1191–229. 10.1093/ehjci/jew190 27880640

[11] Nagueh SF Smiseth OA Appleton CP Byrd BF Dokainish H Edvardsen T. Recommendations for the evaluation of left ventricular diastolic function by echocardiography: an update from the american society of echocardiography and the european association of cardiovascular imaging. Eur Heart J Cardiovasc Img. (2016) 17:1321–60. 10.1093/ehjci/jew082 27422899

[12] Obokata M Kane GC Reddy YNV Olson TP Melenovsky V Borlaug BA. Role of diastolic stress testing in the evaluation for heart failure with preserved ejection fraction. Circulation. (2017) 135:825–38. 10.1161/CIRCULATIONAHA.116.024822 28039229 PMC5330848

[13] Martens P Claessen G Van De Bruaene A Verbrugge FH Herbots L Dendale P. Iron deficiency is associated with impaired biventricular reserve and reduced exercise capacity in patients with unexplained dyspnea. J Card Fail. (2021). 10.1016/j.cardfail.2021.03.010. [Epub ahead of print]. 33838251

[14] Martens P Herbots L Timmermans P Verbrugge FH Dendale P Borlaug BA. Cardiopulmonary exercise testing with echocardiography to identify mechanisms of unexplained dyspnea. J Cardiovasc Transl Res. (2021). 10.1007/s12265-021-10142-8. [Epub ahead of print]. 34110608

[15] Claessen G La Gerche A Voigt J-U Dymarkowski S Schnell F Petit T. Accuracy of echocardiography to evaluate pulmonary vascular and RV function during exercise. J Am Coll Cardiol Img. (2016) 9:532–43. 10.1016/j.jcmg.2015.06.018 26508387

[16] Wolsk E Bakkestrøm R Thomsen JH Balling L Andersen MJ Dahl JS. The influence of age on hemodynamic parameters during rest and exercise in healthy individuals. J Am Coll Cardiol Heart Fail. (2017) 5:337–46. 10.1016/j.jchf.2016.10.012 28017352

[17] Reddy YNV Carter RE Obokata M Redfield MM Borlaug BA. A simple, evidence-based approach to help guide diagnosis of heart failure with preserved ejection fraction. Circulation. (2018) 138:861–70. 10.1161/CIRCULATIONAHA.118.034646 29792299 PMC6202181

[18] Gorter TM Obokata M Reddy YN V Melenovsky V Borlaug BA. Exercise unmasks distinct pathophysiologic features in heart failure with preserved ejection fraction and pulmonary vascular disease. Eur Heart J. (2018) 39:2825–35. 10.1093/eurheartj/ehy331 29947750 PMC6093469

[19] Ha J-W Andersen OS Smiseth OA. Diastolic stress test: invasive and noninvasive testing. J Am Coll Cardiol Img. (2020) 13:272–82. 10.1016/j.jcmg.2019.01.037 31202741

[20] Burgess MI Jenkins C Sharman JE Marwick TH. Diastolic stress echocardiography: hemodynamic validation and clinical significance of estimation of ventricular filling pressure with exercise. J Am Coll Cardiol. (2006) 47:1891–900. 10.1016/j.jacc.2006.02.042 16682317

[21] Drighil A Madias JE Mathewson JW El Mosalami H El Badaoui N Ramdani B. Haemodialysis: effects of acute decrease in preload on tissue doppler imaging indices of systolic and diastolic function of the left and right ventricles. Eur J Echocardiogr. (2008) 9:530–5. 10.1093/ejechocard/jen125 18490307

[22] Cross TJ Kim C-H Johnson BD Lalande S. The interactions between respiratory and cardiovascular systems in systolic heart failure. J Appl Physiol. (2020) 128:214–24. 10.1152/japplphysiol.00113.2019 31774354

[23] Gevaert AB Boen JRA Segers VF Van Craenenbroeck EM. Heart failure with preserved ejection fraction: a review of cardiac and noncardiac pathophysiology. Front Physiol. (2019) 10:638, 1–14. 10.3389/fphys.2019.00638 31191343 PMC6548802

[24] Borlaug BA Olson TP Lam CSP Flood KS Lerman A Johnson BD. Global cardiovascular reserve dysfunction in heart failure with preserved ejection fraction. J Am Coll Cardiol. (2010) 56:845–54. 10.1016/j.jacc.2010.03.077 20813282 PMC2950645

[25] Wang J Fang F Wai-Kwok Yip G Sanderson JE Lee PW Feng W. Changes of ventricular and peripheral performance in patients with heart failure and normal ejection fraction: insights from ergometry stress echocardiography. Eur J Heart Fail. (2014) 16:888–97. 10.1002/ejhf.124 25100109

[26] Masada K Hidaka T Harada Y Kinoshita M Itakura K Higashi A. Mitral systolic velocity at peak exercise predicts impaired exercise capacity in patients with heart failure with preserved ejection fraction. Echocardiography. (2017) 34:217–25. 10.1111/echo.13440 28240427

[27] Wang J Fang F Wai-Kwok Yip G Sanderson JE Feng W Xie J-M. Left ventricular long-axis performance during exercise is an important prognosticator in patients with heart failure and preserved ejection fraction. Int J Cardiol. (2015) 178:131–5. 10.1016/j.ijcard.2014.10.130 25464236

[28] Fitzgerald BT Presneill JJ Scalia IG Hawkins CL Celermajer YM Scalia W. The prognostic value of the diastolic stress test in patients undergoing treadmill stress echocardiography. J Am Soc Echocardiogr. (2019) 32:1298–306. 10.1016/j.echo.2019.05.021 31377071

[29] Huang W Oliveira RKF Lei H Systrom DM Waxman AB. Pulmonary vascular resistance during exercise predicts long-term outcomes in heart failure with preserved ejection fraction. J Card Fail. (2018) 24:169–76. 10.1016/j.cardfail.2017.11.003 29180305

