# Supplementary material for: Exercise Systolic Reserve and Exercise Pulmonary Hypertension Improve Diagnosis of Heart Failure With Preserved Ejection Fraction
Source: Front Cardiovasc Med. 2022 Feb 9;9:814601. doi: 10.3389/fcvm.2022.814601 (PMC8863971; doi:10.3389/fcvm.2022.814601)
Supplement: Supplementary file 1 [file Data_Sheet_1.pdf]

Supplemental Data

Supplemental Methods

Supplemental Figure 1: Representative example of color tissue Doppler image for measuring peak exercise *S'*

Supplemental Figure 2: Receiver operating characteristic curves

Supplemental Figure 3: Decision tree adapted for ASE/EACVI recommendations algorithm.

Supplemental Figure 4: Performance of different DST algorithms for detecting elevated exPAWP

Supplemental Figure 5: Bland-Altman plots of DST parameters.

Supplemental Table 1: Baseline characteristics of the exRHC cohort, stratified according to PAWP ≥ or < 25 mmHg

Supplemental Table 2: Echocardiography, invasive hemodynamics, arterial blood gas measurement and cardiopulmonary exercise test results in the exRHC cohort

Supplemental Table 3: Correlations of echo parameters

References

Supplemental Methods

Cardiopulmonary exercise test (exRHC and DST cohorts)

All patients (both exRHC and DST cohort) underwent a cardiopulmonary exercise test (CPET) with simultaneous acquisition of echocardiographic images. Ventilation (VE), oxygen uptake (VO_2) and carbon dioxide production (VCO_2) were continuously measured through a face mask during exercise. The aerobic threshold was defined as a sustained rise in O_2 ventilatory equivalent, the anaerobic threshold was defined as a sustained rise in CO_2 ventilatory equivalent. Peak VO_2 was defined as the highest 10-second average of VO_2 during exercise [1].

Echocardiography

Echocardiography was performed simultaneously during CPET with Vivid E9 (GE Healthcare) . Measurements were performed offline using EchoPAC software (GE Healthcare) according to current guidelines [2,3]. Peak mitral systolic annular velocity (*S'*) was measured using color tissue Doppler imaging (TDI) at the level of the septal mitral annulus (Supplemental Figure 1, Supplemental File 1). Medial *e'* was measured at the septal mitral valve annulus using pulse wave TDI. In case of E/A fusion, measurements were made before E/A fusion occurred. Systolic pulmonary artery pressure (sPAP) was estimated from TR velocity without adding right atrial pressure. Colloid enhancement of the tricuspid insufficiency signal was systematically employed as previously described [4]. Mean PAP (mPAP) was calculated by the Chemla formula as sPAP*0.61+2. Stroke volume (SV) and cardiac output (CO) were calculated using the left ventricular outflow tract method.

In 22 patients, measurements were repeated twice in a blinded fashion by 3 observers to assess intra-observer variability (ABG, cardiologist specialized in echocardiography; JV, cardiologist specialized in echocardiography; and a cardiologist in training).

Invasive hemodynamic exercise test

In the exRHC cohort only, a pulmonary artery catheter (Edwards Lifesciences) was placed under fluoroscopic guidance at the catheterization lab before start of the CPET and the right radial artery was cannulated with a 5F arterial catheter. The fluid filled catheters were then connected to a pressure transducer unit (PowerLab, ADInstruments) with zeroing at the mid axillary level. Every 3 minutes during exercise and at peak exercise, arterial and mixed venous blood gas samples were obtained and PAWP was measured. Other hemodynamic measurements were registered continuously. Hemodynamic tracings were stored in LabChart v8.1 (ADInstruments) for offline analysis by an experienced cardiologist blinded to echocardiographic measurements (J.V.). All pressure measurements were performed at end-expiration by averaging at least 3 cardiac cycles. CO was calculated using the Fick method.

Sample size calculation

Using f test power calculation for repeated measures (GPower v3.1.9), we estimated that a sample of 16 patients would provide 90% power to detect a difference in echocardiographic parameters between elevated and normal PAWP. We used an effect size of 0.86, based on the difference in

exE/e' between patients with invasively proven HFpEF and non-cardiac dyspnea in the study by Obokata et al [5].

Statistical analysis

Continuous variables were expressed as mean ± standard deviation or median (interquartile range) in case of a skewed distribution. Categorical variables were expressed as percentages. Baseline comparisons were performed using Mann-Whitney-U test, Pearson's Chi-squared test or Fisher's Exact test where appropriate. Comparisons between 3 groups were performed using Kruskal-Wallis test with Dunn test for between-group comparisons (continuous variables), and Pearson's Chi-squared test with pairwise nominal independence test (categorical variables). Interobserver variability was calculated using a two-way agreement intra-class correlation model and using Bland-Altman plots. Correlations were assessed using Spearman's rho.

DST parameters were compared between patients with elevated vs. normal exPAWP using Mann-Whitney-U test (single measurement during DST, for example mPAP/CO slope) or linear mixed models (repeated measurement during DST, for example *E/e'*). Linear mixed models were constructed using patient number as random factor, and exercise, elevated exPAWP, and their interaction as fixed factors. For each DST parameter with potential to identify elevated exPAWP, a receiver operating characteristic curve was determined, and area under the curve (AUC) was calculated with the trapezoidal rule. 95% confidence intervals (CI) were calculated using stratified bootstrap replicates. AUC were compared using Delong's test.

Holm method was used as correction for multiple comparisons. A two-sided p-value <0.05 was considered significant. All data was analyzed using R v3.6.3 (R Foundation for Statistical Computing) with packages *FSA, irr, multcomp, nlme, pROC, plotROC, rcompanion,* and *tidyverse.*

Supplemental Figure 1: Representative example of color tissue Doppler image for measuring peak exercise *S'*. Peak exercise *S'* was 8 cm/s in this patient. This was measured at the 4th cardiac cycle in this recording (red line). Note that the first steep upstroke in the tissue Doppler signal (red arrow) should NOT be measured. Also note the small variability of *S'* compared to the large variability in *e'* at peak exercise.

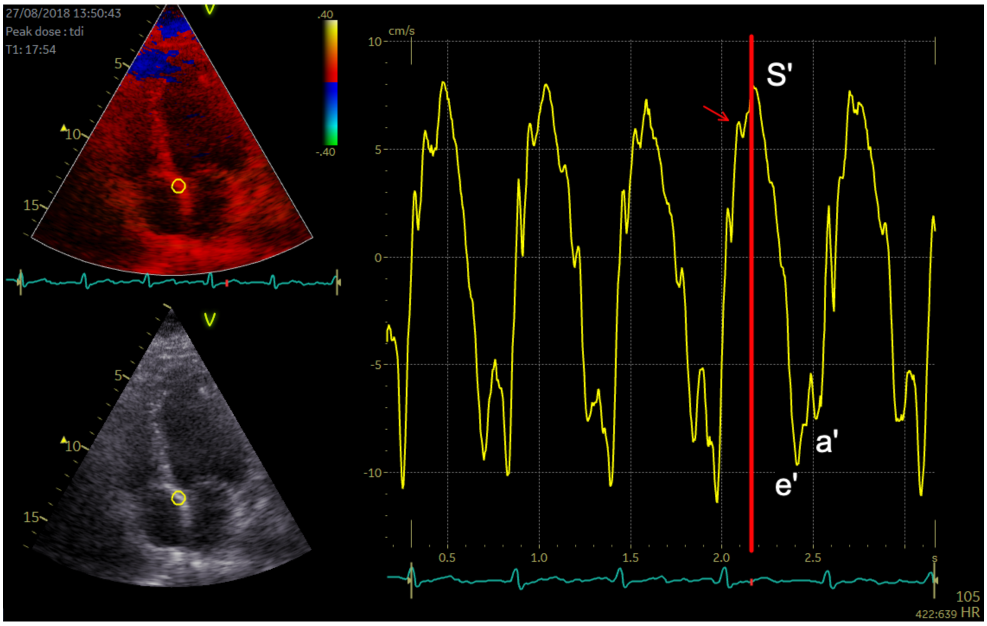

Supplemental Figure 2: Receiver operating characteristic curves. Receiver operating characteristic curves for identifying elevated exPAWP using *exS'* (red), mPAP/CO slope (green), peak CI (brown), septal *exE/e'* (blue) and peak sPAP (grey). AUC, area under the receiver operating characteristic curve, CI, cardiac index; CO, cardiac output; exE/e', highest septal E/e' recorded during exercise; exS', S' at peak exercise; mPAP, mean pulmonary artery pressure; sPAP, systolic pulmonary artery pressure.

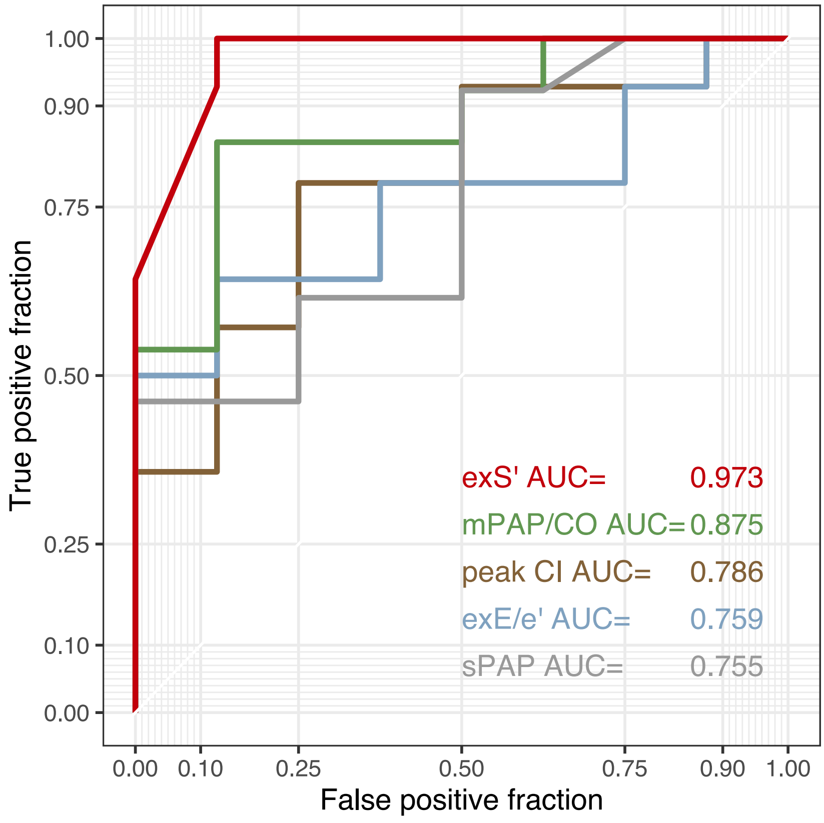

Supplemental Figure 3 (next page): Decision tree adapted for ASE/EACVI recommendations algorithm.

A: Derivation of the decision tree in the exRHC cohort. Step 1: the existing approach in ASE/EAVCI recommendations is maintained. Step 2: *exS'* and mPAP/CO slope are determined, probability of HFpEF is considered high if *exS'* <9.5 cm/s and mPAP/CO slope ≥3.2 mmHg/L. Thus all patients with exPAPW ≥25 mmHg are identified. A single patient is false positive using this approach. **B:** Application of the decision tree to the non-invasive DST cohort. In step 1, the existing approach in ASE/EAVCI recommendations is maintained. Of 243 patients with inconclusive results according to ASE/EACVI recommendations, 97 (40%) had *exS'* >9.5 cm/s without exercise pulmonary hypertension, we propose that probability of HFpEF is low in these patients. A total of 45 patients (19% of inconclusive results) had *exS'* <9.5 cm/s with elevated mPAP/CO slope suggesting exercise pulmonary hypertension. We propose that probability of HFpEF is high in these patients. In the remaining patients, we propose to perform additional investigations before establishing a diagnosis of HFpEF. CO, cardiac output; exE/e', highest septal E/e' recorded during exercise; exS', S' at peak exercise; mPAP, mean pulmonary artery pressure; TR, tricuspid regurgitation velocity.

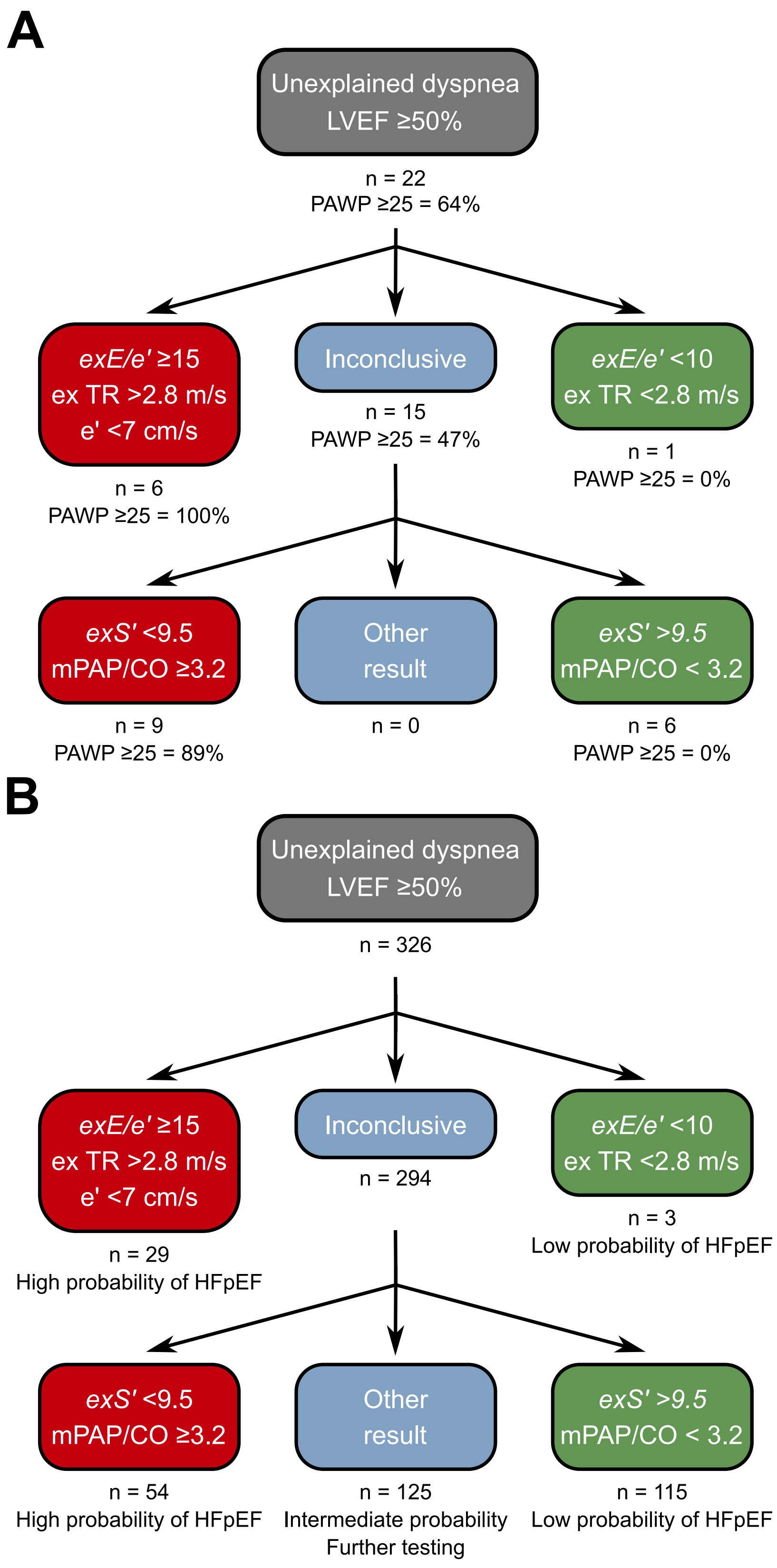
A
Unexplained dyspnea
LVEF ≥50%
n = 22
PAWP ≥25 = 64%
exE/e' ≥15
ex TR >2.8 m/s
e' <7 cm/s
n = 6
PAWP ≥25 = 100%
Inconclusive
n = 15
PAWP ≥25 = 47%
exE/e' <10
ex TR <2.8 m/s
n = 1
PAWP ≥25 = 0%
exS' <9.5
mPAP/CO ≥3.2
n = 9
PAWP ≥25 = 89%
Other
result
n = 0
exS' >9.5
mPAP/CO < 3.2
n = 6
PAWP ≥25 = 0%
B
Unexplained dyspnea
LVEF ≥50%
n = 326
exE/e' ≥15
ex TR >2.8 m/s
e' <7 cm/s
n = 29
High probability of HFpEF
Inconclusive
n = 294
exE/e' <10
ex TR <2.8 m/s
n = 3
Low probability of HFpEF
exS' <9.5
mPAP/CO ≥3.2
n = 54
High probability of HFpEF
Other
result
n = 125
Intermediate probability
Further testing
exS' >9.5
mPAP/CO < 3.2
n = 115
Low probability of HFpEF

Supplemental Figure 4: Performance of different DST algorithms for detecting elevated exPAWP. True positives: correct diagnosis of HFpEF in patients with elevated exPAWP. False positives: wrong diagnosis of HFpEF in patients with normal exPAWP. Our proposed decision tree (Figure 4) noninvasively detects 100% of true positives at the cost of 13% false positives. ASE/EACVI recommendations propose to diagnose HFpEF if septal *exE/e'* is ≥15, baseline *e'* is <7 cm/s, and exercise tricuspid regurgitation is >2.8 m/s [10,11]. The HFA consensus proposes stricter thresholds: exercise TR >3.4 m/s and average *exE/e'* ≥15 [8]. *ExE/e'* alone considers septal *exE/e'* ≥15. These approaches all detect ≤50% of true positives. ASE/EACVI, American Society of Echocardiography/European Association of Cardiovascular Imaging; HFA, Heart Failure association; exE/e', highest septal E/e' recorded during exercise.

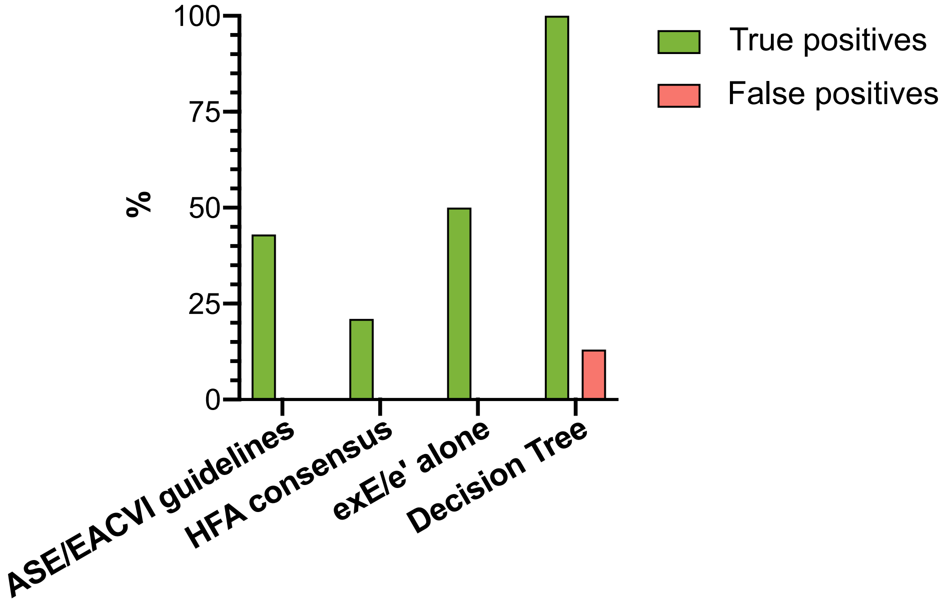

Supplemental Figure 5: Bland-Altman plots of key echocardiographic parameters. Difference vs average plot of key echocardiographic parameters for 22 patients of the DST cohort and 3 observers. **A:** *exS'* as cm/s, note that some data points are overlapping. **B:** mPAP/CO as mmHg•L^{-1}•min^{-1} **C**: peak mPAP as mmHg, calculated by the Chemla formula as sPAP*0.61+2 with sPAP estimated from colloid-enhanced tricuspid regurgitation signal without adding right atrial pressure, **D:** peak CO as L•min^{-1}, calculated by the left ventricular outflow tract method, **E:** peak E/e' ratio. Dashed lines: upper and lower limits of agreement, full line: mean difference (bias). CO = cardiac output, *exS'* = peak exercise septal systolic velocity on color Doppler, mPAP = mean pulmonary artery pressure, SD = standard deviation, sPAP = systolic pulmonary artery pressure.

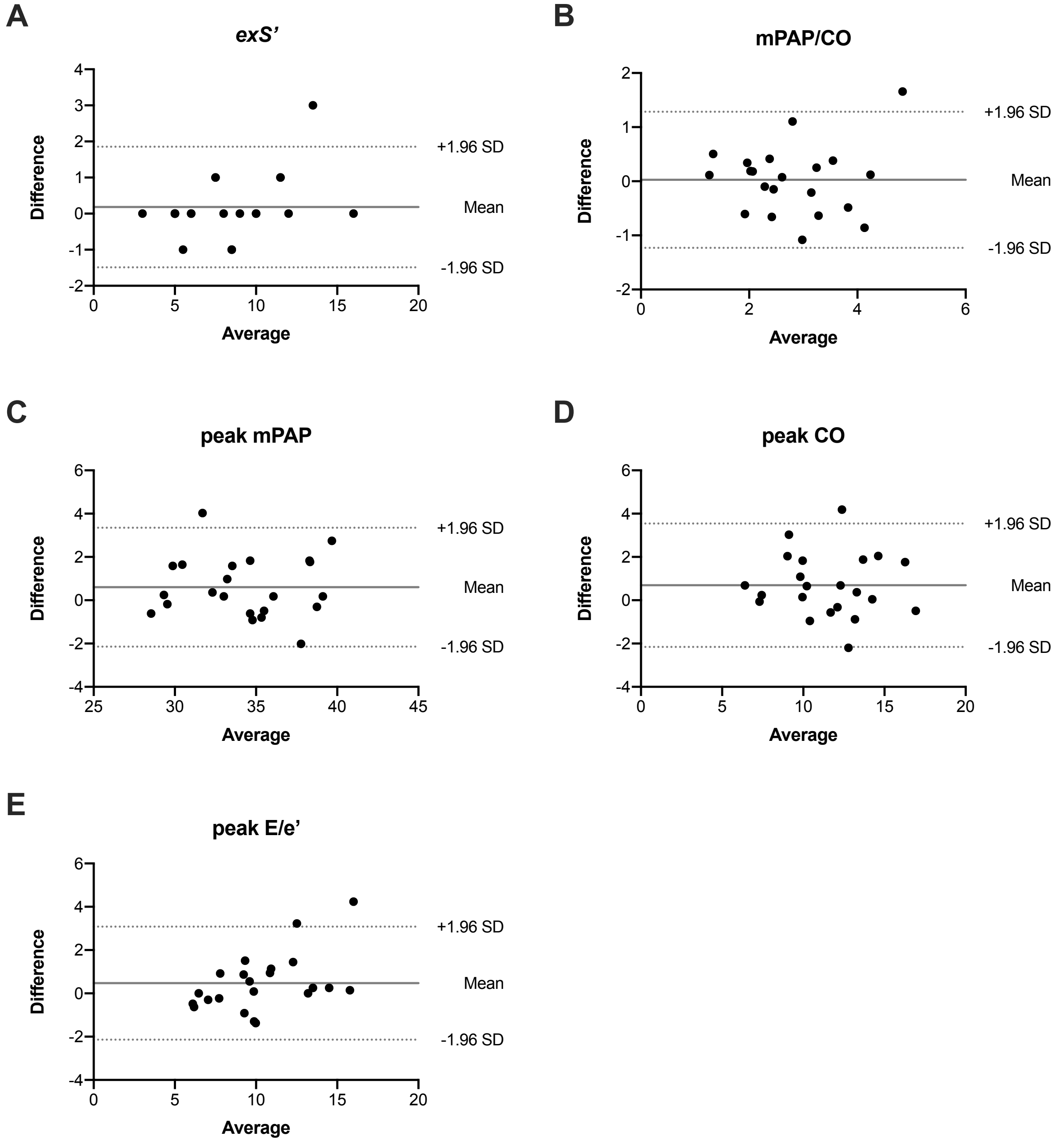

Supplemental Table 1: Baseline characteristics of the exRHC cohort, stratified according to PAWP ≥ or < 25 mmHg

**Characteristic**	**PAWP ≥25 mmHg (n=14)**	**PAWP <25 mmHg (n=8)**	**P value**
Age, median (IQR), years	67 (64-73)	59 (55-62)	**.027**
Female sex, no. (%)	7 (50)	3 (38)	.675
Heart rate, median (IQR), bpm	66 (59-71)	72 (70-78)	**.044**
Systolic blood pressure, median (IQR), mmHg	151 (137-156)	128 (127-151)	.673
BMI, median (IQR), kg/m^2	28.2 (26.2-31.8)	26.7 (25.0-28.1)	.238
**Past medical history**			
Atrial fibrillation, no. (%)	4 (29)	1 (13)	.736
Coronary heart disease, no. (%)	8 (57)	0 (0)	**.018**
Diabetes, no. (%)	2 (14)	1 (13)	.999
Hypertension, no. (%)	9 (64)	2 (25)	.183
Valvular heart disease, no. (%)	1 (7)	0 (0)	.999
**Medication use**			
ACE inhibitor or ARB, no. (%)	6 (42)	0 (0)	.051
Aldosterone antagonist, no. (%)	4 (29)	0 (0)	.254
Beta blocker, no. (%)	10 (71)	1 (13)	**.024**
Calcium antagonist, no. (%)	3 (21)	1 (13)	.999
Diuretic, no. (%)	4 (29)	1 (13)	.613
Nitrate, no. (%)	2 (14)	0 (0)	.515
**Laboratory analysis**			
Hemoglobin, median (IQR), g/dL	13.6 (13.3-14.4)	15.0 (14.6-16.2)	.147
EGFR, median (IQR), mg/dL	68 (65-72) *(n=8)*	75 (72-102)	**.021**

P value from Mann-Whitney-U test (continuous variables) or Fisher's exact test (categorical variables). ACE = angiotensin conversion enzyme, ARB = angiotensin receptor blocker, BMI = Body mass index, EGFR = Estimated glomerular filtration rate using CKD-EPI formula.

Supplemental Table 2: Echocardiography, invasive hemodynamics, arterial blood gas measurement and cardiopulmonary exercise test results in the exRHC cohort

Echocardiography	Elevated exPAWP		Normal exPAWP		P-Group	P-Exercise	P-Inter-action	P Peak vs. Peak
	Rest	Peak	Rest	Peak				
E/e', median (IQR) *	11.1 (9.0-15.9)	14.7 (11.9-18.4)	8.4 (7.4-9.6)	11.1 (9.0-12.9)	.090	.049	.798	**.043**
S', median (IQR), cm/s	4.0 (3.1-4.8)	5.5 (5.0-8.0)	6.0 (5.0-7.2)	12.0 (10.0-12.8)	.025	.002	.002	**<.001**
Systolic PAP, median (IQR), mmHg	21 (18-24)	59 (45-69)	20 (18-22)	43 (32-56)	.834	<.001	.026	**.005**
LV ejection fraction, median (IQR), %	66 (59-70)	74 (58-78)	62 (60-64)	70 (60-72)	.571	.049	.857	.999
LV end diastolic volume index, median (IQR), mL/m²	48 (40-55)	46 (41-50)	44 (41-52)	39 (36-46)	.516	.212	.623	/
Cardiac index, median (IQR), L/min/m²	2.3 (2.2-3.6)	5.1 (4.4-5.8)	2.6 (2.5-2.9)	6.1 (5.8-6.5)	.884	<.001	.017	**.026**
RV fractional area change, median (IQR), %	54 (50-59)	56 (52-65)	50 (49-58)	55 (53-63)	.778	.180	.929	/
LV mass index, median (IQR), g/m²	90 (75-116)		62 (55-74)		**.006**	/	/	/
Left atrial volume index, median (IQR), mL/m²	19.8 (14.9-24.6)		17.5 (16.7-27.2)		.827	/	/	/
Mean PAP/CO slope, median (IQR), mmHg/L	4.8 (3.9-5.9)		2.5 (1.1-3.3)		**.003**	/	/	/
CO/VO_2 slope, median (IQR)	5.4 (4.6-6.3)		4.9 (3.6-6.8)		.547	/	/	/
**Invasive hemodynamics**	**Elevated exPAWP**		**Normal exPAWP**		**P-Group**	**P-Exercise**	**P-Inter-action**	**P Peak vs. Peak**
	**Rest**	**Peak**	**Rest**	**Peak**				
Heart rate, median (IQR), bpm	70 (59-83)	121 (114-136)	71 (69-83)	144 (128-158)	.779	<.001	.022	.086

Systolic blood pressure, median (IQR), mmHg	168 (150-193)	214 (196-235)	155 (150-163)	206 (184-236)	.476	.001	.837	.964
PAWP, median (IQR), mmHg	13 (10-19)	32 (30-42)	9 (7-10)	20 (18-22)	.071	<.001	.007	**<.001**
Systolic PAP, median (IQR), mmHg	32 (30-37)	73 (66-79)	22 (21-26)	50 (46-57)	.139	<.001	.032	**<.001**
Right atrial pressure, median (IQR), mmHg	8 (4-11)	18 (16-25)	4 (3-4)	9 (9-12)	.063	<.001	.044	**<.001**
Diastolic pressure gradient, median (IQR), mmHg	1 (0-2)	5 (3-11)	1 (1-5)	8 (4-12)	.773	.012	.275	.401
Cardiac index, median (IQR), L/min/m^2	2.2 (1.9-2.5)	5.1 (4.3-6.1)	2.5 (2.2-3.2)	6.5 (6.0-8.3)	.271	<.001	.534	.167
Pulmon. vascular resistance, median (IQR), dynes*s/cm^5	136 (102-176)	123 (97-200)	114 (63-129)	107 (51-125)	.200	.704	.961	/
Systemic vascular resistance, median (IQR), dynes*s/cm^5	2111 (1809-2640)	907 (629-1262)	1687 (1282-1953)	652 (545-710)	.030	<.001	.289	.648
PAWP/CO slope, median (IQR), mmHg/L	3.4 (1.5-5.6)		1.2 (1.0-1.6)		**.016**	/	/	/
Mean PAP/CO slope, median (IQR), mmHg/L	6.0 (3.7-8.5)		2.4 (2.0-3.3)		**.006**	/	/	/
CO/VO$_2$ slope, median (IQR)	9.4 (8.2-11.5)		6.7 (5.3-9.2)		.165	/	/	/
**Arterial blood gas measurement**	**Elevated exPAWP**		**Normal exPAWP**		**P-Group**	**P-Exercise**	**P-Inter-action**	**P Peak vs. Peak**
	**Rest**	**Peak**	**Rest**	**Peak**				
Central venous O$_2$ saturation, median (IQR), %	65 (63-67)	38 (32-44)	69 (69-70)	32 (27-36)	.372	<.001	.060	.198
O$_2$ extraction, median (IQR), mL/dL	5.5 (5.1-6.0)	10.6 (8.0-12.2)	5.2 (4.9-5.6)	12.3 (11.5-14.0)	.599	<.001	.031	.084
Lactate, median (IQR), mmol/L	1.1 (0.9-1.3)	5.2 (4.3-6.1)	1.0 (0.9-1.3)	5.7 (3.2-7.6)	.968	<.001	.069	**.026**
**Cardiopulmonary exercise test**	**Elevated exPAWP**		**Normal exPAWP**		**P-Group**	**P-Exercise**		

	**Rest**	**Peak**	**Rest**	**Peak**			**P-Interaction**	**P Peak vs. Peak**
VO_2,<br>median (IQR), mL/kg/min	3.1<br>(2.6-3.7)	12.5<br>(10.9-14.4)	3.7<br>(3.2-3.8)	22.7<br>(17.8-24.0)	.610	<.001	.003	**<.001**
Workload,<br>median (IQR), W	0<br>(0-0)	90<br>(71-111)	0<br>(0-0)	157<br>(109-183)	.999	<.001	.008	**<.001**
Respiratory exchange ratio,<br>median (IQR)	0.77<br>(0.70-0.81)	1.13<br>(1.08-1.16)	0.86<br>(0.82-0.86)	1.15<br>(1.11-1.21)	.049	<.001	.611	.338
Breathing reserve,<br>median (IQR), %	100<br>(100-100)	47<br>(29-56)	100<br>(100-100)	61<br>(54-72)	.999	<.001	.776	.664
VE/VCO_2 slope,<br>median (IQR)	31.8<br>(28.7-35.3)		25.4<br>(23.3-26.8)		**.013**	/	/	/

Elevated exPAWP was defined as PAWP ≥25 mmHg during peak exericse. P values from linear mixed models analysis (2 time points) or Mann-Whitney-U test (single time point). P Peak vs Peak from Holm-corrected post hoc multiple comparisons. CO = cardiac output, exPAWP = pulmonary artery wedge pressure during peak exercise, LV = left ventricular, PAWP = pulmonary artery wedge pressure, PAP = pulmonary artery pressure, PAWP = pulmonary artery wedge pressure, VE = ventilation, VCO_2 = carbon dioxide removal, VO_2 = oxygen uptake, * Highest *E/e'* value obtained during exercise.

Supplemental Table 3: Correlations of echo parameters

	exS'	*exE/e'*	**Peak sPAP**	**mPAP/CO slope**	**Peak cardiac index**	**LV mass index**
exS'	/	Rho = -.42<br>p <.001	Rho = -.08<br>p = .162	Rho = -.43<br>p <.001	Rho = .46<br>p <.001	Rho = -.02<br>p = .724
exE/e'	/	/	Rho = .20<br>p <.001	Rho = .39<br>p <.001	Rho = -.33<br>p <.001	Rho = .17<br>p = .002
**Peak sPAP**	/	/	/	Rho = .53<br>p <.001	Rho = -.01<br>p = .887	Rho = .17<br>p = .002
**mPAP/CO slope**	/	/	/	/	Rho = -.63<br>p <.001	Rho = .05<br>p = .355
**Peak cardiac index**	/	/	/	/	/	Rho = -.03<br>p = .630
**LV mass index**	/	/	/	/	/	/

Spearman's rho and corresponding P value. Although some correlations were statistically significant, none of the parameters demonstrated a very strong correlation (rho >0.7). This supports the separate use of *exE/e'*, *exS'* and mPAP/CO in the interpretation of diastolic stress testing. CI = cardiac output, *exE/e'* = early mitral inflow velocity over early diastolic annular velocity during exercise, *exS'* = peak exercise septal systolic velocity, LV = left ventricular, mPAP = mean pulmonary artery pressure, sPAP = systolic pulmonary artery pressure.

References

1 Guazzi M, Arena R, Halle M, Piepoli MF, Myers J, Lavie CJ. 2016 focused update: clinical recommendations for cardiopulmonary exercise testing data assessment in specific patient populations. *Eur Heart J* 2018; **39**: 1144–1161.

2 Nagueh SF, Smiseth OA, Appleton CP, Byrd BF, Dokainish H, Edvardsen T, *et al.* Recommendations for the Evaluation of Left Ventricular Diastolic Function by Echocardiography: An Update from the American Society of Echocardiography and the European Association of Cardiovascular Imaging. *Eur Heart J Cardiovasc Img* 2016; **17**: 1321–1360.

3 Lang RM, Badano LP, Mor-Avi V, Afilalo J, Armstrong A, Ernande L, *et al.* Recommendations for Cardiac Chamber Quantification by Echocardiography in Adults: An Update from the American Society of Echocardiography and the European Association of Cardiovascular Imaging. *J Am Soc Echocardiogr* 2015; **28**: 1-39.e14.

4 Claessen G, La Gerche A, Voigt J-U, Dymarkowski S, Schnell F, Petit T, *et al.* Accuracy of Echocardiography to Evaluate Pulmonary Vascular and RV Function During Exercise. *J Am Coll Cardiol Img* 2016; **9**: 532–543.

5 Obokata M, Kane GC, Reddy YNV, Olson TP, Melenovsky V, Borlaug BA. Role of Diastolic Stress Testing in the Evaluation for Heart Failure With Preserved Ejection Fraction. *Circulation* 2017; **135**: 825–838.

6 Reddy YNV, Carter RE, Obokata M, Redfield MM, Borlaug BA. A Simple, Evidence-Based Approach to Help Guide Diagnosis of Heart Failure With Preserved Ejection Fraction. *Circulation* 2018; **138**: 861–870.
